# Psychometric Evidence for Indirect Assessment of Child Abuse Risk in Child Welfare-Involved Mothers

**DOI:** 10.3390/children9050711

**Published:** 2022-05-12

**Authors:** Christina M. Rodriguez, Paul J. Silvia

**Affiliations:** 1 Department of Psychology, University of Alabama at Birmingham, Birmingham, AL 35294-1170, USA; 2 Department of Psychology, University of North Carolina at Greensboro, Greensboro, NC 27402-3170, USA; p_silvia@uncg.edu

**Keywords:** analog assessment, case-control studies, child abuse, implicit measurement, indirect assessment, psychometrics, social information processing theory

## Abstract

Most research on factors related to physical child abuse risk rely heavily on direct self-report measures, which is a methodological strategy susceptible to participant response distortions. Such methodological reliance obfuscates the interpretations rendered about the risk factors predictive of child abuse. Efforts to develop alternative indirect assessment approaches, such as analog tasks, show promise, although most of those studies have applied these methods to community samples rather than with child welfare-involved samples. The present study evaluated the psychometric evidence for four separate analog tasks that have not yet been considered with mothers identified for child maltreatment by child welfare services, also contrasted to a sociodemographically matched sample of mothers. The results indicate acceptable reliability for the analog tasks, with additional evidence of validity. However, the two groups of mothers did not substantively differ across measures, suggesting that identification for abuse through child protective services does not differentiate from those closely matched on critical sociodemographic characteristics. The promising preliminary results of these analog tasks in the current study suggest that indirect analog assessment approaches to estimate child abuse risk could be useful in efforts to minimize dependence on self-report methods.

## 1. Introduction

Apart from its moral implications, child maltreatment incurs exorbitant costs to society as a consequence of compromised productivity, physical and mental health burden, and macrosystem responses, amounting to an estimated $7 trillion annual cost worldwide [1]. In the U.S., nearly four million children are investigated for maltreatment every year, but only a fraction (less than a fifth) meet the high standards required for substantiation, with varying criteria in different jurisdictions [2]. Physical abuse, which typically requires physical acts that injure a child, is the second most common form of child maltreatment after neglect [2], comprising over a quarter of cases of maltreatment [3]. 

Nonetheless, the evidence is overwhelming that reports through official channels capture only a fraction of child maltreatment. Based on estimates from the last congressionally mandated report in the U.S.—which surveys professionals who routinely interact with children and observed demonstrable harm—the rate of physical abuse is 2.5 times the rate of substantiated cases through child protective services [3], with similar findings in the Netherlands [4]. Phone surveys on physical abuse from nationally representative samples in the U.S. inquiring about physical injury in the prior year suggest exponentially higher rates than even those in the congressional report [5]. Consequently, the World Health Organization estimates that as many as 18% of U.S. children have been subject to physical abuse [6], most never reported to protective services. Those parents identified by child protective services potentially reflect families living in under-resourced communities who experience more surveillance of their parenting that subsequently comes to the attention of officials [7]. 

Because so much child maltreatment remains unreported to officials, researchers have developed proxy measures to capture the attitudes and behaviors that characterize parents who may become abusive, a concept termed *child abuse risk* [8,9,10]. For example, parents who engage in any form of physical parent-child aggression (PCA) pose a risk of escalating in intensity to abuse [11,12,13], whereby more frequent use of PCA is a prominent predictor of child abuse. Assessment of child abuse risk permits potential identification of those who have not been identified through official channels yet share commonalities with those who have been substantiated for abuse. Rather than conceptualize measures of parents’ child abuse risk as forensic tools to establish whether child abuse has transpired, assessment of child abuse risk factors is valuable for child abuse prevention. The ability to estimate child abuse risk can inform target areas to incorporate into child abuse prevention programs, particularly those efforts committed to primary, universal prevention and/or secondary prevention [14] seeking to avert child abuse in the first place. 

An array of qualities contributes to heightened child abuse risk, but one of the leading theories to describe the cognitions characterizing those who become abusive is based in Social Information Processing (SIP) theory [15,16]. As applied to PCA, SIP theory proposes that parents hold pre-existing beliefs (e.g., approval of using PCA for discipline) which predisposes them to its use. When confronted with a discipline encounter, four stages unfold: a parent may first misperceive the situation due to poor attention or over-reactivity (Stage 1), then develop adverse interpretations about their child’s behavior (Stage 2), inadequately consider their alternative explanations and options (Stage 3), and ultimately fail to monitor their administration of PCA (Stage 4). 

The current literature indicates that parents who hold pre-existing beliefs that endorse PCA use as a discipline technique are predisposed toward higher risk for abuse [17,18,19,20]. Poor emotional regulation and frustration tolerance can interfere with accurate perceptions of a discipline encounter (Stage 1), and such problematic regulatory abilities have been linked to increased child abuse risk [20,21,22,23]. Furthermore, parents may also attribute negative intent to perceived child misbehavior (Stage 2), which is a robust predictor of elevated child abuse risk [24,25,26,27,28]. 

However, largely due to convenience, this evidence base overwhelming relies on direct, explicit assessment approaches—namely, self-report questionnaires—to measure concepts relevant to abuse risk. This reliance is problematic because parents may intentionally (or unconsciously) misrepresent themselves in a favorable manner. Indeed, this overreliance on explicit self-report is a well-recognized bias in the extant literature in child abuse risk, acknowledged in the limitations of most current empirical work. Alternatively, indirect assessment approaches using analog methods are designed to measure a construct in a manner analogous to the construct (through behavior simulations or implicit means) wherein the respondent is unaware of the intent and/or scoring of the measure [29,30]. Analog tasks themselves vary to the extent to which their purpose is explicit to the participant; compared to less implicit (more explicit) analog tasks, more implicit measures tend to obtain lower effect sizes in their association with explicit self-report measures [30,31,32]. Indirect assessment strategies are well regarded as less biased approaches [30,33], which enable multimethod assessment of constructs [30] and provide meaningful insights [32]. In particular, indirect assessment approaches can be useful for topics where social desirability (and legal) concerns may be prominent, such as constructs related to child abuse risk [29,31]. 

Despite the field’s reliance on self-report measures, some indirect assessment approaches have been developed directly pertaining to constructs relevant to child abuse risk, some of which were considered in a recent review of implicit measures [29]. A substantial majority of the studies reviewed involved comparisons of groups recruited from the community who evidenced lower versus higher child abuse risk using proxy measures of abuse risk [29]. Of the few studies including parents officially identified as abusive through child welfare services, several report no significant differences from comparison groups not identified as abusive on a number of dimensions relevant to abuse risk [34,35,36], although other studies suggest some group differences [29]. However, much of that literature is now dated, as researchers have increasingly opted for the convenience of self-report methods. Although such group differences involving substantiated samples of parents may not be reliably observed, in groups at increased child abuse risk, indirect approaches do appear to demonstrate convergent validity with explicit measures and concurrent validity with traditional measures of child abuse risk [29]. Indirect measures may thus be useful adjuncts to traditional self-report measures, given the benefits of multimethod assessment protocols [37,38]. 

Therefore, the current study evaluated the reliability, concurrent, and convergent validity of four separate analog measures relevant to child abuse risk that have never been administered to a sample of mothers identified through child protective services for child maltreatment. Analog measures evaluated in this study focused on the following constructs: (1) approval of PCA, an SIP pre-existing schema; (2) frustration tolerance, which could interfere with SIP Stage 1 processing; (3) negative child intent attributions, a feature of SIP Stage 2 processing, and (4) an indirect measure of child abuse risk pertaining to inclination to use PCA. In addition to evaluating psychometrics properties with the child welfare-involved sample, this group was contrasted with a sociodemographically comparable group of mothers. Beyond considering potential group differences, we also considered whether the pattern of concurrent and convergent evidence was more apparent in the child welfare-involved mothers because they represented the highest child abuse risk.

## 2. Materials and Methods

### 2.1. Participants 

The current study involved two samples of mothers. The first sample was comprised of 38 child welfare-involved mothers participating in the “Advancing Innovative Methods to Study Parenting” (AIMS-P) study, conducted in Birmingham, Alabama, USA. Mothers had been indicated/substantiated for child maltreatment by the county child protective services; mothers identified by child welfare services involving child physical abuse are mandated to designated parent training programs. AIMS-P mothers identified for these concerns were recruited either directly from child welfare or through these specific training programs.

Using a matched case-control design with a 2-to−1 matching ratio, the second sample involved 76 mothers drawn from the “Following First Families Study” (Triple-F) study, a four-wave longitudinal study tracking factors contributing to child abuse risk across time, also conducted in Birmingham, Alabama, USA. Half of the 203 families recruited demonstrated one or more sociodemographic risk factors (i.e., ≤150% of the federal poverty line, receipt of public assistance, ≤high school education, single parenthood, ≤age 18); none had documented histories of engaging in child maltreatment. These primiparous mothers were recruited from ob/gyn clinics during their last trimester in the pregnancy and followed for four waves until their child was age 4–4½ years.

To create a comparison sample, Triple-F data from two mothers were extracted to match each AIMS-P mother on race; income group; educational attainment group; receipt of public assistance; and maternal age ±3 years. Participants selected their annual income group from the following: <$3000, $3000–7999, $8000–12,999, $13,000–19,999, $20,000–29,999, $30,000–39,999, $40,000–49,999, $50,000–59,999, $60,000–69,999, $70,000–79,999, $80,000–99,999, $100,000+. Participants selected their educational level from the following groups: grade school, some high school, some college/technical school, completed 4-year college, some post-college schooling, completed post-graduate degree. The two samples were comparable in their sociodemographic matching criteria (see Table 1) but given that the Triple-F sample involved first-time mothers, AIMS-P mothers had more children and older children than Triple-F mothers (both variables included in the covariate set described below). 

### 2.2. Procedures 

Both samples were collected in person, with all measures delivered by using Inquisit 4.0 software and participants wearing headphones. In questions pertaining to their own children, mothers were instructed to focus on the target child (in AIMS-P, their most challenging child; in Triple-F, the child born into the study). Mothers provided written informed consent, in which participants were assured confidentiality. The University of Alabama at Birmingham Institutional Review Board approved both studies.

### 2.3. Measures

#### 2.3.1. PCA Approval

The Parent-Child Aggression Acceptability Movie Task (Parent-CAAM) [39] is an analog task designed to assess approval of PCA. The task includes eight 90-s video clips from films that include a scene of PCA of varying intensity (five clips of physical abuse, three clips of physical discipline). In the development of the Parent-CAAM, clips were categorized as abuse or discipline anonymously by social workers. The Parent-CAAM presents the clips in randomized order and respondents were asked to stop the scene if and when they judge the scene has become abusive. Deliberating on whether a scene is abusive would delay responding; thus, the number of milliseconds until the respondent stops the scene was recorded. Parent-CAAM total scores are averaged across video clips, with higher scores indicative of greater PCA approval. Across multiple samples who have not been identified for child abuse, Parent-CAAM scores have evidenced reliability as well as concurrent and convergent validity [39].

The Adult-Adolescent Parenting Inventory (AAPI-2, Version A), Value of Corporal Punishment Scale (an alternate version than utilized for PCA risk below) includes 11 items on the acceptability of physical discipline [8]. Each item is rated on a 5-point scale from 1 (strongly disagree) to 5 (strongly agree), which are summed across items to create a total score wherein higher scores indicated greater approval for use of PCA. This subscale has demonstrated reliability and concurrent validity with reported parenting [40].

#### 2.3.2. Frustration Tolerance

The Frustration Intolerance Task (FIT) [41] is an analog task intended to assess frustration tolerance in a parent-relevant scenario. A computer simulation presents a two-dimensional visual maze of grocery store aisles, with a practice trial to learn how to navigate the maze using the arrow keys. The parent is then asked to imagine they are in a grocery store and need to exit because their child has become distressed. Although selection of the arrow keys implies they are moving through the store, no exit is possible. The parent is instructed to continue searching for an exit throughout the unsolvable maze but on the screen is a large “STOP” button, should they choose to quit. The current study utilized the 10-min version that includes an audio overlay of a child experiencing a temper tantrum for the duration of their efforts to seek an exit. The number of seconds until quitting was captured, with longer duration suggesting greater frustration tolerance. Across multiple samples of varying levels of risk (other than parents identified as abusive), lower FIT scores relate to child abuse risk, greater use of PCA, and increased heart rate [41].

The Frustration Discomfort Scale (FDS) [42] is a self-report measure of tolerance to frustration and discomfort. Participants were asked to rate seven items on a 5-point scale from 1 (strongly disagree) to 5 (strongly agree). Item scores were summed for a total score, oriented such that higher total scores suggest poor frustration tolerance. The FDS demonstrates acceptable reliability and differentiates clinical from comparison samples [42].

#### 2.3.3. Negative Intent Attributions

The Noncompliance Implicit Association Test (N-IAT) [43] is an implicit measure similar to the original IAT task [44]. Respondents sort terms describing child behavior (e.g., “temper tantrum”, “follow directions”) into “good/bad” and “obey/disobey” categories. Attitudes that are consistent with implicit beliefs are typically sorted more quickly than those inconsistent with beliefs. After a series of randomized trials, participants received a difference score based on their response times to “obey-bad” versus “obey-good” conditions. Smaller difference (*d*) scores reflect more negative behavior attributions. Smaller N-IAT *d*-scores have been observed to relate to self-reported negative attributions and abuse risk in samples of parents not identified as abusive [43].

The Plotkin Child Vignettes (PCV) [45] include 18 vignettes describing child behavior that can be perceived as misbehavior. Parents were asked to indicate the extent to which they judge the child’s behavior in the scene as intentionally provocative on a 9-point scale from 1 (did not mean to annoy me at all) to 9 (the only reason the child did this was to annoy me). Scores are summed wherein higher total scores suggest more negative attributions. Higher scores have been observed in parents identified as abusive [46] and have been related to implicit measures of negative attributions [27].

#### 2.3.4. Child Abuse Risk

The Response Analog to Child Compliance Task (ReACCT) [47] is an analog task intended to simulate a realistic parent-child interchange. In the scene, the parent is unexpectedly late getting their child to school and needs to direct their child in order to leave home. In each of a series of steps, the parent reportedly provides the child instructions, and the child is depicted as either complying or not complying with that directive. Although the parent provides 12 total instructions to the child, a total of 20 steps are presented as the parent can remain “stuck” in a step if the child continues to not comply. For each of these steps, parents can select from 16 options how they would react to the child’s behavior. Adaptive responses (e.g., for compliance) receive positive weighted values whereas maladaptive responses (e.g., PCA) receive negative weighted values. During the entire task, the parent hears and sees a timer to induce time urgency. Parents are instructed that they receive bonuses for their child’s timely compliance (50 cent bonus for each compliance). ReACCT scores can be computed for Noncompliance (12 items) and Compliance (8 items), with higher scores indicative of greater child abuse risk. The current study focused on ReACCT noncompliance scores, which have previously been related to traditional measures of child abuse risk as well as abusive discipline tactics in samples with varying levels of abuse risk but not in parents identified for abuse [47]. 

The Child Abuse Potential Inventory (CAPI) [48] is the most frequently used measure developed to identify elevated child abuse risk. The CAPI provides respondents with 160 Agree/Disagree statements, although only 77 of the items are extracted and variably weighted to provide an Abuse Scale score. The Abuse Scale does not assess parenting and instead focuses on parents’ perceptions of distress from significant intrapersonal and interpersonal difficulties and rigidity, qualities that have been observed in parents who are abusive toward children. Higher Abuse Scale scores suggest greater child abuse risk. Multiple studies have demonstrated strong psychometric properties for the CAPI Abuse Scale, with scores further demonstrating predictive validity in correctly classifying those substantiated for abuse versus their counterparts [10].

The Adult-Adolescent Parenting Inventory-2 (AAPI-2) [8] focuses on parenting beliefs and behaviors selected to distinguish between maltreating and non-maltreating samples. The AAPI-2 includes 40 items using a 5-point scale from 1 (strongly disagree) to 5 (strongly agree), which was summed across items and oriented such that higher total scores indicated greater child abuse risk. Prior research has supported both reliability and validity of the AAPI-2 [40].

### 2.4. Analytic Plan

Preliminary statistics were performed with SPSS 27. Cronbach’s alpha was computed for internal consistency reliability estimates for measures involving multiple items. We considered potential covariates for subsequent analyses if they evidenced even marginal associations with any of the outcome measures at *p* < 0.10. Analyses of covariance (ANCOVAs) were conducted to evaluate sample differences. Partial correlations were then performed by sample, controlling for needed covariates. Fisher *r*-to-*z* transformations with one-tailed tests determined if the difference in magnitude of the correlations was stronger for the AIMS-P sample relative to the Triple-F sample.

## 3. Results

### 3.1. Preliminary Coviariate Analyses

Given strong associations between income and education, we created a composite socioeconomic status (SES) score with standardized values to minimize multicollinearity concerns. Examination of potential sociodemographic covariates indicated that parental age, target child age, number of children, and SES were not associated with the four analog tasks, with the exception of a significant association between the N-IAT and SES (r = 0.22, *p* < 0.05) and between parental age and ReACCT Noncompliance (r = −0.24, *p* < 0.05). For the comparator self-reports, one or more of these sociodemographic factors significantly related to the FDS, Plotkin Attribution, AAPI-2 Total score, and CAPI Abuse Scale scores. Thus, the full set of sociodemographic covariates were utilized given their potential effects on the self-report measures.

### 3.2. Reliability and Sample Group Differences

The two analog tasks for which coefficient alpha is relevant (Parent-CAAM, ReACCT) demonstrated acceptable levels of internal consistency reliability for both groups (see Table 2). ANCOVAs comparing groups, with covariates, are also presented in Table 2, indicating that only the CAPI Abuse Scale score showed sample group differences. (Note, the findings are comparable to *t*-test analyses without covariates).

### 3.3. Validity Correlations

With regard to convergent validity evidence (see Table 3), the Parent-CAAM was significantly related to the comparator measure for self-reported PCA approval on the AAPI-2 subscale for both samples. Although this magnitude was stronger for the AIMS-P sample of mothers, who were identified through child welfare, compared to the community-recruited matched sample of Triple-F mothers, this magnitude difference was not statistically significant. For the FIT, the association with the comparator self-report measure for frustration tolerance was significantly more strongly related in the AIMS-P sample compared to the Triple-F sample (*z* = 1.92, *p* = 0.027). Regarding negative attributions, the AIMS-P mothers’ N-IAT *d*-scores were significantly related to the comparator self-report measure of negative child intent attributions, which was not observed in the Triple-F sample. Likely due to limited power, the magnitude of these associations for the N-IAT did not significantly differ between samples. Finally, with regard to ReACCT Noncompliance scores in relation to comparator measures of abuse risk, although the Triple-F sample evidenced a significant association with one of the self-report measures (AAPI-2), the magnitudes of the correlations did not substantively differ between samples (and indeed, in the AIMS-P sample, the effect of ReACCT with the CAPI Abuse Scale appeared stronger than that observed in the Triple-F sample).

Turning to concurrent validity of the analog tasks with abuse risk measures (also Table 3), the Parent-CAAM demonstrated associations with the AAPI-2 Total score and CAPI Abuse score comparable between both groups, with the notable exception that the Parent-CAAM demonstrated a more robust effect with the ReACCT score in the AIMS-P sample (although not significantly stronger than that observed for the Triple-F sample, likely due to power). The FIT demonstrated significant associations with all three measures of abuse risk (AAPI-2, CAPI, ReACCT) for the AIMS-P sample, whereas for the Triple-F sample, the only significant association observed was with the AAPI-2. Indeed, the effect of the FIT with the CAPI Abuse Scale was significantly stronger for the AIMS-P sample than with the Triple-F sample (*z* = 1.78, *p* = 0.037). For the N-IAT, scores for the AIMS-P sample were significant with the ReACCT, and significantly stronger than those observed with the Triple-F sample (*z* = 2.36, *p* = 0.009); although the CAPI was significantly related to N-IAT scores in the Triple-F sample, the effect was not significantly different than that observed in the AIMS-P sample. Notably, the three analog measures not directly measuring child abuse risk (PCA approval, frustration tolerance, negative child attributions) of Parent-CAAM, FIT, and N-IAT all demonstrated significant relations with the ReACCT task for the AIMS-P sample (effects not observed for the Triple-F sample).

## 4. Discussion

The present investigation evaluated the psychometric evidence for four separate analog tasks with a sample of mothers identified/substantiated for child maltreatment, including a comparison to a sociodemographically matched sample of mothers. The two analog measures for which internal consistency would be an appropriate measure of reliability (Parent-CAAM and ReACCT) both demonstrated acceptable and comparable reliability in both samples of mothers. The samples did not significantly differ in their mean scores on either the analog tasks or the self-report measures, with the exception that the child welfare-involved sample scored higher on the Child Abuse Potential Inventory. The pattern of correlations indicates that three of the four analog measures (pertaining to PCA approval, frustration tolerance, and negative child attributions) demonstrated convergent validity in the child welfare-involved sample, with some indication of stronger effects for the mothers who had been identified by child protective services. Although the fourth analog measure (ReACCT) did not evidence substantial convergent validity with the two self-report measures of child abuse risk in either sample, this abuse risk measure was consistently more evidently associated with the other analog measures, particularly for mothers in the child welfare-involved sample.

Based on prior research on constructs related to Social Information Processing (SIP) theory [15,26], this study focused on PCA approval (potential pre-existing schema), frustration tolerance (potentially affecting Stage 1 processing), and negative child attributions (potential Stage 2 interpretation). The three analog measures of these constructs were all significantly related to self-report measures for the highest risk sample of mothers identified through child protective services, with weaker or no effects observed for the matched sample. Of the three constructs, the analog measure of low frustration tolerance, which has previously been observed to relate to child abuse risk in a variety of community samples [22,23,41], demonstrated convergent and concurrent validity for the child welfare-involved sample relative to the matched sample. The pattern of findings also supported convergent validity of the Parent-CAAM and N-IAT analog measures of PCA approval and negative child attributions, respectively, particularly for the mothers in the maltreatment group, consistent with previously observed relations from self-reports of PCA approval [17,18,19] and negative attributions [24,25,26]. The evidence for concurrent validity of these two analog tasks in relation to child abuse risk is more modest, particularly when considering relations to traditional self-report measures of abuse risk. Although the Parent-CAAM and N-IAT analog tasks were not as consistently related to the self-report proxy measures of child abuse risk for either group of mothers, the substantive effect sizes for the analog measure of abuse risk (ReACCT) were more notable, particularly for the mothers identified through child welfare services. In other words, the indirect assessment measures were interrelated despite employing very different modalities in these four analog tasks. These findings underscore the importance of considering the links of these SIP constructs with child abuse risk without relying exclusively on self-report approaches. The observed relations in earlier work may reflect self-report method biases that would benefit from continued work using multimethod assessments [38].

Interestingly, the current findings suggest that those substantiated for child maltreatment may not substantively differ from sociodemographically comparable parents living in the same communities. Prior work suggested that analog tasks do not consistently distinguish parents identified for maltreatment from comparison samples [29,34,35,36]. The current findings extend this observation, indicating that the highest risk sample generally did not statistically differ from the sociodemographically matched sample on either self-report or analog measures. The one exception to this finding involved the Child Abuse Potential Inventory, a measure that heavily emphasizes personal distress rather than constructs more directly related to parenting [10]. The sample of mothers indicated or substantiated for child maltreatment were mandated to these parenting programs and could have been experiencing additional distress from the possibility of losing custody of their child. In contrast, none of the other measures tapped psychological distress, and thus may account for the lack of differences between the sociodemographically comparable groups.

Indeed, prior work has identified that those who are indicated/substantiated for maltreatment in four different sites in the U.S. were indistinguishable from those who were reported but not substantiated on multiple developmental or behavioral indices [49]. Longitudinal studies also suggest that using official substantiation to identify vulnerable families appears to overlook children and families in need [50]. Consequently, some have argued that families with unsubstantiated reports of maltreatment should also receive services [51,52]. In effect, our findings suggest that mothers indicated/substantiated for maltreatment do not substantially differ from mothers residing in the same communities when controlling for sociodemographics. Given the potential that some under-resourced communities are more closely monitored than others [7], the few families that are referred and then substantiated by child protective services may simply reflect a selection of parents drawn from their communities, but the label of substantiation is what some have termed as a “distinction without a difference” [49]. Clearly child maltreatment is not limited to those in low-income communities, nor are all those within such communities inevitably likely to abuse. Developing additional tools to screen for important factors related to child abuse risk is critical. Such strategies could be applied more widely to identify individual parents who may not intend to harm their children yet struggle with parenting—and who may not conform to a stereotyped profile of an abusive parent.

Continued research is clearly needed on these constructs and these samples, particularly using research designs that address some of the current study limitations. Foremost among these limitations is the limited sample size of the child welfare-involved group, followed by the focus exclusively on mothers. Future research should consider a larger sample size of indicated/substantiated child welfare-involved parents inclusive of fathers and adopting a case-control design that better matches the groups on the age and number of children. Such a replication with a larger sample could also consider additional aspects of psychometric evidence, such as predictive and incremental validity, as well as longitudinal work that could evaluate stability of the analog test scores in child welfare-involved samples. Furthermore, additional validation with other measures of child abuse risk [53] as well as other methodologies in relation to analog tasks, could be considered, such as parent-child observational measures. Finally, greater understanding of the reasoning behind parents’ use of PCA is needed because different racial groups, for example, may be motivated to utilize PCA for different reasons [54], and such differences might affect their responses to implicit or behavioral analog tasks.

## 5. Conclusions

Indirect assessment approaches in the form of analog tasks can provide promising adjunct, and potentially more objective, strategies to assess constructs that are particularly vulnerable to participant response distortions, such as factors relevant to child abuse risk. The current findings suggest that more work should consider utilizing these types of assessment alternatives in order to clarify whether the presumptive risk factors are, indeed, predictive of child abuse risk rather than reflecting method biases. Each of the analog tasks considered in the current evaluation demonstrated preliminary evidence as a promising means to assess constructs theorized to elevate physical child abuse risk. As our understanding of these risk factors increases, the ability to tailor strategies to modify such factors improves. The development of alternative approaches to estimate child abuse risk is particularly meaningful for efforts intended to prevent maltreatment, both in terms of primary and secondary prevention, which could identify a wider array of parents who could benefit from services.

## Figures and Tables

**Table 1:** Demographics by Study Sample.

	AIMS-P	Triple-F	X^2^ or *t*-Test
	*n* (%) or *M* (*SD*)	*n* (%) or *M* (*SD*)	
Race			X^2^ = 0.25
Black	28 (73.7%)	57 (75.0)	
White	9 (23.7%)	18 (23.7%)	
Black Bi-racial	1 (2.6%)	1 (1.3%)	
Public Assistance			X^2^ = 0.26
Yes	32 (84.2%)	61 (80.3%)	
No	6 (15.8%)	32 (19.7%)	
Single Parent			X^2^ = 1.75
Yes	22 (57.9%)	34 (43.4%)	
No	16 (42.1%)	42 (56.6%)	
Mother Age (years)	29.54 (5.68)	27.66 (5.21)	*t* = 1.75
Annual Income Group	3.42 (2.40)	3.33 (2.11)	*t* = 0.21
Educational Level	3.11 (1.06)	3.42 (1.00)	*t* = 1.56
Child Age	2.54 (1.60)	6.33 (4.23)	*t =* 5.28 ***
Number Other Children	0.30 (0.54)	2.9 (1.23)	*t* = 12.66 ***

*Note*. *** *p* < 0.001.

**Table 2:** Sample Descriptive Statistics and Group Differences.

	AIMS-P		Triple-F		
	α ^†^	*M* (*SD*)	α	*M* (*SD*)	*F*
AAPI-2 Corp Punish	0.77	26.47 (7.64)	0.77	29.71 (8.64)	0.67
Parent-CAAM	0.88	16.91 (13.58)	0.76	17.50 (12.88)	0.31
FDS	0.87	18.16 (7.07)	0.93	17.86 (7.49)	0.14
FIT		245.53 (200.85)		235.27 (180.80)	0.86
Plotkin Attribution	0.77	36.89 (14.62)	0.91	40.43 (21.94)	1.14
Noncompliance-IAT		1.02 (0.45)		0.89 (0.53)	2.19
AAPI-2 Total	0.85	101.87 (18.23)	0.86	103.36 (20.31)	0.27
CAPI Abuse Scale		187.71 (104.81)		109.72 (70.64)	6.03 *
ReACCT Noncomply	0.78	−2.97 (12.44)	0.79	0.49 (14.36)	1.04

*Note*. ANCOVA covariates of maternal age, child age, number of children, socioeconomic status. AAPI-2 = Adult-Adolescent Parenting Scale-2; Parent-CAAM = Parent-Child Aggression Acceptability Movie Task; FDS = Frustration Discomfort Scale; FIT = Frustration Intolerance Task; IAT = Implicit Association Test *d*-scores; CAPI = Child Abuse Potential Inventory; ReACCT = Response Analog to Child Compliance Task. ^†^ Coefficient alpha not computed for single scores (FIT, N-IAT) or variably weighted CAPI Abuse Scale Score. * *p* < 0.05.

**Table 3:** Partial Correlations by Sample Group.

	Parent-CAAM		FIT		N-IAT		ReACCT	
	AIMS-P	Triple-F	AIMS-P	Triple-F	AIMS-P	Triple-F	AIMS-P	Triple-F
Self-Report ^a^	0.40 *	0.30 *	−0.45 **	−0.09	−0.34 *	−0.14		
AAPI-2	0.24	0.28 *	−0.36 *	−0.27 *	−0.11	−0.17	0.26	0.32 **
CAPI	0.19	0.14	−0.36 *	0.01	−0.08	−0.24 *	0.27	0.10
ReACCT	0.40 *	0.22	−0.36 *	−0.22	−0.49 **	0.05		

*Note*. Partial correlations control for maternal age, child age, number children, socioeconomic status. Parent-CAAM = Parent-Child Aggression Acceptability Movie Task; FIT = Frustration Intolerance Task; N-IAT = Noncompliance Implicit Association Test; ReACCT = Response Analog to Child Compliance Task, Noncompliance Scale; AAPI-2 = Adult-Adolescent Parenting Scale-2; CAPI = Child Abuse Potential Inventory, Abuse Scale. ^a^ Parent-CAAM self-report = AAPI-2 Corporal Punishment Subscale; FIT self-report = Frustration Discomfort Scale; N-IAT self-report = Plotkin Child Vignettes, Attribution Scale. * *p* < 0.05, ** *p* < 0.01.

## Data Availability

The data analyzed in this study are available upon request from the corresponding author. The data are not publicly available in accordance with the stipulations of the Alabama Department of Human Resources (Child Welfare Services Division) study approval, in accordance with the consent provided by participants, and as approved by the University of Alabama at Birmingham Institutional Review Board.
